# Patient-Reported Quality of Life Outcomes after Moderately Hypofractionated and Normofractionated Proton Therapy for Localized Prostate Cancer

**DOI:** 10.3390/cancers14030517

**Published:** 2022-01-20

**Authors:** Koichiro Nakajima, Hiromitsu Iwata, Yukiko Hattori, Kento Nomura, Kensuke Hayashi, Toshiyuki Toshito, Yukihiro Umemoto, Shingo Hashimoto, Hiroyuki Ogino, Yuta Shibamoto

**Affiliations:** 1 Department of Radiation Oncology, Nagoya Proton Therapy Center, Nagoya City University West Medical Center, Nagoya 462-8508, Japan; h-iwa-ncu@nifty.com (H.I.); hattoriy@med.nagoya-cu.ac.jp (Y.H.); knomura01i04m08t@gmail.com (K.N.); hogino2@med.nagoya-cu.ac.jp (H.O.); 2 Department of Proton Therapy Technology, Nagoya Proton Therapy Center, Nagoya 462-8508, Japan; k.hayashi.12@west-med.jp; 3 Department of Proton Therapy Physics, Nagoya Proton Therapy Center, Nagoya 462-8508, Japan; t.toshitou.20@west-med.jp; 4 Department of Urology, Nagoya City University West Medical Center, Nagoya 462-8508, Japan; uro-ume@med.nagoya-cu.ac.jp; 5 Department of Radiology, Nagoya City University Graduate School of Medical Sciences, Nagoya 467-8601, Japan; hashimoto.ncu@gmail.com (S.H.); yshiba@med.nagoya-cu.ac.jp (Y.S.); 6 Department of Radiation Oncology, Narita Memorial Proton Center, Toyohashi 441-8021, Japan

**Keywords:** proton therapy, prostate cancer, moderately hypofractionation, quality of life, EPIC-50

## Abstract

**Simple Summary:**

For patients with localized prostate cancer (PCa), information on the quality of life (QOL) after treatment is important when determining their preferred treatment option. In external beam radiation therapy, moderate hypofractionation (MH) is becoming an alternative standard for PCa treatment and MH is increasingly used in proton therapy (PT). Although MHPT is a promising strategy, there is little evidence regarding the data of long-term QOL after MHPT. This study evaluated patient-reported QOL over three years after MHPT and compared the data with that after normofractionated PT (NFPT) using the Expanded Prostate Cancer Index Composite-50. We revealed urinary QOL was temporarily decreased with clinically meaningful changes at 1 month, but did not observe clinically meaningful QOL deterioration in other assessment points in the urinary, bowel, and sexual domains over three years after MHPT. In addition, the QOL after MHPT and NFPT was similar overall.

**Abstract:**

We retrospectively evaluated the three-year patient-reported quality of life (QOL) after moderately hypofractionated proton therapy (MHPT) for localized prostate cancer in comparison with that after normofractionated PT (NFPT) using the Expanded Prostate Cancer Index Composite-50. Patients who received MHPT (60–63 Gy (relative biological effectiveness equivalents; RBE)/20–21 fractions) (*n* = 343) or NFPT (74–78 Gy (RBE)/37–39 fractions) (*n* = 296) between 2013 and 2016 were analyzed. The minimum clinically important difference (MCID) threshold was defined as one-half of a standard deviation of the baseline value. The median follow-up was 56 months and 83% completed questionnaires at 36 months. Clinically meaningful score deterioration was observed in the urinary domain at 1 month in both groups and in the sexual domain at 6–36 months in the NFPT group, but not observed in the bowel domain. At 36 months, the mean score change for urinary summary was −0.3 (MHPT) and −1.6 points (NFPT), and that for bowel summary was +0.1 and −2.0 points; the proportion of patients with MCID was 21% and 24% for urinary summary and 18% and 29% for bowel summary. Overall, MHPT had small negative impacts on QOL over three years, and the QOL after MHPT and NFPT was similar.

## 1. Introduction

Various treatment strategies exist for localized prostate cancer (PCa) and the cure rate does not vary greatly among them [1]. Therefore, information on the quality of life (QOL) after treatment is important for patients when determining their preferred treatment option [2,3]. Recent technological advances have led to the emergence of multiple external beam radiation therapy (EBRT) strategies, including proton therapy (PT), for localized PCa [4,5,6]. Although excellent overall survival and biochemical failure-free survival have been reported, data on patient-reported outcomes such as disease-specific QOL after each EBRT strategy are not sufficient, especially regarding PT.

Hypofractionated EBRT is widely used in current PCa treatments [7]. The general advantages of hypofractionation include improved patient convenience and resource utilization. Biologically, it may provide an additional advantage due to the low α/β ratio of PCa [8,9]. Furthermore, recent high conformal techniques enable dose escalation while minimizing unnecessary doses to healthy tissues. Thus, hypofractionation with high conformal RT techniques is expected to improve the efficacy with less patient harm.

Several large-scale trials using modern X-ray RT (XRT) techniques reported favorable outcomes of moderate hypofractionation (MH, typically defined by a fraction size between 2.4–3.4 Gy [7]) [10,11,12]. Sub-analyses of these studies revealed a similar long-term (two years or more) QOL to that after normofractionation (NF) [13,14,15]. In this context, MH with high conformal RT techniques is becoming an alternative standard for PCa treatment and MH is also increasingly used in PT [16,17,18]. However, studies investigating the long-term outcomes of MHPT are limited, and in particular, there is little evidence regarding the data of long-term QOL [16,17]. Therefore, the purpose of the present study was to evaluate the three-year patient-reported QOL after MHPT with 3 Gy (relative biological effectiveness equivalents; RBE) per fraction and to compare it with that after NFPT with 2 Gy (RBE) per fraction.

## 2. Materials and Methods

### 2.1. Study Design

This study retrospectively analyzed disease-specific QOL data of patients with localized PCa who underwent PT at Nagoya Proton Therapy Center between February 2013 and December 2016. We started NFPT in February 2013, and MHPT in October 2014. After the start of MHPT, patients could choose either NFPT or MHPT. This retrospective study was conducted to compare the three-year patient-reported QOL after NFPT and MHPT, and was approved by the institutional review board of Nagoya City Hospital (21-04-342-17). Eligibility criteria were (1) histologically confirmed PCa; (2) T1–T3N0M0 disease according to the 7th edition of TNM staging of the Union for International Cancer Control; (3) Eastern Cooperative Oncology Group Performance status of 0–2; (4) age >20 years; (5) no previous RT to the pelvis; (6) no prior surgery for PCa; and (7) written informed consent.

### 2.2. Treatment Protocols

The NFPT schedule was 74 Gy (RBE) in 37 fractions for low-risk patients, and 78 Gy (RBE) in 39 fractions for intermediate- and high-risk patients. The MHPT schedule was 60 Gy (RBE) in 20 fractions for low-risk patients, and 63 Gy (RBE) in 21 fractions for intermediate- and high-risk patients. The risks were categorized according to National Comprehensive Cancer Network (NCCN) Risk Categories [1]. We classified very low- and low-risk patients as “low-risk patients”, and high- and very high-risk patients as “high-risk patients” throughout this study. All irradiation was delivered once a day with five fractions a week. The RBE value for our proton beams was determined to be 1.1 [19].

In principle, low-risk patients received no androgen-deprivation therapy (ADT), intermediate-risk patients received 6-month neoadjuvant ADT, and high-risk patients received two-year neoadjuvant and adjuvant ADT. These ADT schedules were not applicable to patients who previously received ADT at the initial visit. Combined androgen blockade was recommended for ADT, but monotherapy with an LH-RH analogue or an antiandrogen was allowed.

### 2.3. Proton Therapy

Our treatment planning and delivery procedures were previously described in detail [20,21]. Briefly, most patients were implanted with two gold fiducial markers in the prostate and underwent computed tomography (CT) simulation. Pelvic magnetic resonance imaging was performed for fusion with the CT images. No rectal spacers were used. The clinical target volume (CTV) included the prostate only for low-risk patients, the prostate and proximal one-third volume of the seminal vesicles (SV) for intermediate-risk patients, and the prostate and proximal half of the SV for high-risk patients. Dose constraints are summarized in Table S1. All treatments consisted of right and left lateral beam arrangements with incident proton beam energies mainly from 145 to 225 MeV. A passive scattering technique was used in most cases, but a spot-scanning technique was also used. For passive scattering beam treatments, the CTV was expanded to create a planning target volume (PTV) with craniocaudal and anterior 6-mm, posterior 5-mm, and 9–12-mm lateral margins [22]. For spot-scanning treatments, the definition of a beam-specific PTV reported by Park et al. [23] was used for expansion of the CTV with craniocaudal and anterior 6-mm, posterior 5-mm, and 7–10-mm lateral margins [24]. Patients were encouraged to fill their bladders to 150–250 mL. A purgative, mainly magnesium oxide, was prescribed to most patients for rectum emptying, and when excessive air was present in the rectum, it was removed by a flexible catheter. After positioning, two sets of orthogonal digital radiographs were obtained for daily image-guided treatment delivery. PT planning and treatment were performed with VQA and PROBEAT III (Hitachi, Ltd., Tokyo, Japan).

### 2.4. Measurement of Patient-Reported QOL

Patient-reported QOL data were collected within 1 month before the start of PT (baseline), and at follow-up visits 1, 6, 12, and 36 months after completion of PT. The QOL data were scored using the Expanded Prostate Cancer Index Composite (EPIC)-50 [25]. The EPIC instrument transforms the patient responses to questions to a scale from 0 to 100, with higher scores representing better QOL. In this study, three domains of EPIC scores were analyzed: urinary, bowel, and sexual domains.

### 2.5. Statistical Analysis

The baseline characteristics and EPIC scores of the patients were compared between the NFPT and MHPT groups using Student’s t-test or the Mann–Whitney U test for continuous variables and Fisher’s exact test for categorical variables. A mixed-effect model for repeated measures was applied for regression analysis of changes in EPIC scores from baseline after PT, and least square means at 1, 6, 12, and 36 months were cross-sectionally compared between the NFPT and MHPT groups. To evaluate clinically meaningful changes, the minimum clinically important difference (MCID) was applied; the threshold was defined as half of a standard deviation (SD) for each domain or subscale baseline score in all patients [26] because an anchor-based evaluation for each subscale score was absent. The numbers of patients whose score deterioration reached the MCID threshold were counted, and the proportions were compared between the NFPT and MHPT groups using Fisher’s exact test. In addition, answers to specific questions in urinary and bowel domains were analyzed. The proportion of patients who did not have a moderate to large problem at baseline and who subsequently developed a moderate to large problem at each follow-up point was calculated. All statistical tests were two-sided and a *p* value < 0.05 was considered to be significant. All calculations were performed using EZR [27], which is based on R and R Commander (R Foundation for Statistical Computing, Vienna, Austria).

## 3. Results

### 3.1. Baseline Patient Characteristics and QOL Scores, and Data Collection

A total of 296 patients treated using NFPT and 343 patients treated using MHPT were analyzed. Although three patients developed biochemical or clinical progression and nine died during follow-up within 36 months after PT, they were not excluded from this study. The median follow-up period was 56 months (IQR, 47–64) on the date of QOL data extraction (15 March 2020). Baseline patient characteristics are presented in Table 1. There were no significant differences between the NFPT and MHPT groups regarding age, T stage, Gleason score, NCCN risk category, prostate volume, CTV volume, comorbidity of hypertension and diabetes, or ADT usage. The initial PSA was higher in the NFPT group than in the MHPT group (median (IQR); 9.0 (6.1–14.8) vs. 7.6 (5.6–12.3), *p* = 0.016). Spot-scanning beams were used more often in NFPT than in MHPT (32 patients (11%) vs. 1 patient (0.3%), *p* < 0.001). There were no significant differences in mean baseline EPIC scores (Table 2).

The compliance rates for the EPIC questionnaire were 100%/100% (NFPT/MHPT) at baseline, 98%/96% at 1 (IQR, 1–1) month, 97%/92% at 6 (IQR, 6–7) months, 96%/92% at 12 (IQR, 12–13) months, and 87%/79% at 36 (IQR, 36–37) months. The EPIC assessment was considered completed when at least one of the domains was scored. Passive scattering and spot-scanning techniques produced slightly different dose distributions in the surrounding normal tissues such as the rectum, bladder, femoral head, and obturator muscle. However, a previous study suggested no differences in QOL between the two techniques [22], and in this study, no QOL scores differed between the patients treated by the two techniques; therefore, they were analyzed together.

### 3.2. Urinary Domain

**Figure 1:**
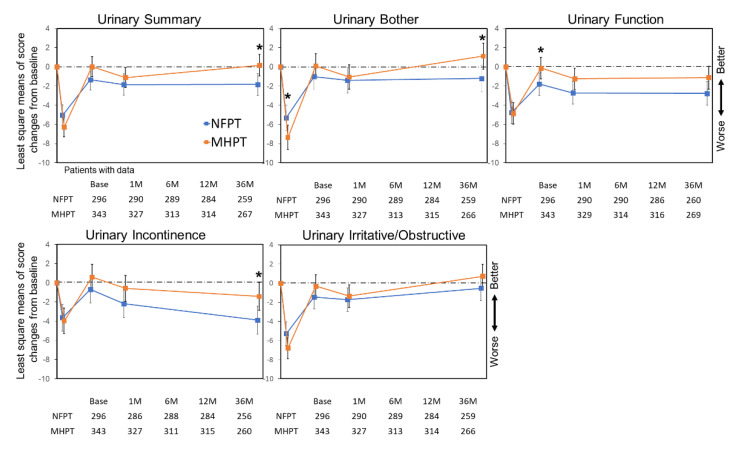
Score changes in EPIC urinary domains. Least square means of score changes from baseline are plotted. Time-points represent baseline, 1, 6, 12, and 36 months, from the left. Error bars represent the 95% confidence interval. Asterisks (*) indicate significant differences (*p* < 0.05) between NFPT and MHPT. Abbreviations: NFPT, normofractionated proton therapy; MHPT, moderately hypofractionated proton therapy.

Changes in urinary domain scores from baseline are shown in Figure 1. Mean score deteriorations beyond the MCID threshold were observed only at 1 month in the following scales: urinary summary (−5.0 points), function (−4.8), and irritative/obstructive (−5.3) in the NFPT group, and urinary summary (−6.2), bother (−7.3), function (−4.9), and irritative/obstructive (−6.8) in the MHPT group (Table 3). The proportion of patients who experienced MCID for urinary bother was higher in the MHPT group (169/327, 52%) than in the NFPT group (121/290, 42%) at 1 month (*p* = 0.015). At 36 months, the proportion in the NFPT vs. MHPT group was 24% (62/259) vs. 21% (57/268), *p* = 0.47 for urinary summary; 28% (73/259) vs. 18% (49/266), *p* = 0.010 for bother; 26% (67/260) vs. 21% (56/269), *p* = 0.18 for function; 25% (65/256) vs. 19% (49/260), *p* = 0.089 for incontinence; and 22% (56/259) vs. 18% (47/266), *p* = 0.27 for irritative/obstructive. Statistically significant differences in mean score changes between the NFPT and MHPT groups were detected for urinary bother at 1 month (−5.0 vs. −7.3; *p* = 0.029), urinary function at 6 months (−1.9 vs. −0.1; *p* = 0.045), and urinary summary, bother, and incontinence at 36 months (−1.6 vs. −0.3; *p* = 0.014, −0.7 vs. 1.1; *p* = 0.019 and −3.6 vs. −1.5; *p* = 0.018, respectively) (* in Figure 1).

**Figure 2:**
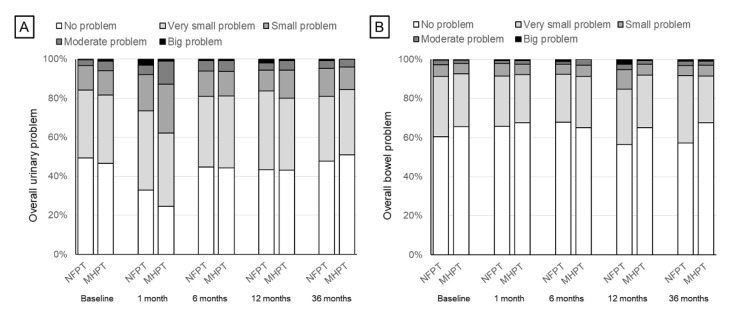
Specific questions in EPIC for overall urinary and bowel bother. (**A**): “Overall, how big a problem has your urinary function been for you during the last 4 weeks?” (**B**): “Overall, how big a problem have your bowel habits been for you during the last 4 weeks?”.

Regarding individual items, the proportion of patients who reported moderate to large problems for overall urinary function at baseline was 3.4% and 5.8% in the NFPT and MHPT groups, respectively. At 1 month, the proportion increased to 7.9% and 12.7%, respectively, whereas it decreased to and remained around 5% at subsequent time points (Figure 2). Upon analysis of specific symptom questions, moderate–large problems for urinary frequency during the day were reported more frequently in the MHPT group than in the NFPT group at 1 month (13% vs. 7.6%; *p* = 0.04), whereas moderate–large problems for urinary leakage were more often reported in the NFPT group than in the MHPT group at 12 and 36 months (2.5% vs. 0.3%; *p* = 0.031 and 2.3% vs. 0%; *p* = 0.014, respectively) (Table S2).

### 3.3. Bowel Domain

**Figure 3:**
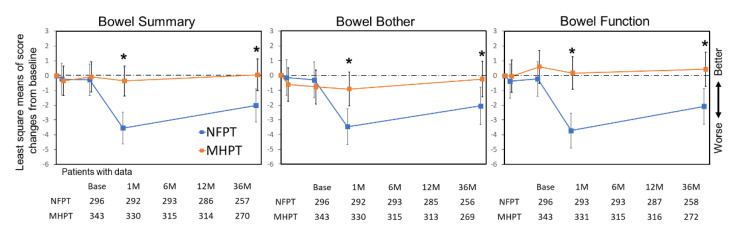
Score changes in EPIC bowel domains. Least square means of score changes from baseline are plotted. Time-points represent baseline, 1, 6, 12, and 36 months, from the left. Error bars represent the 95% confidence interval. * indicate significant differences (*p* < 0.05) between NFPT and MHPT. Abbreviations: NFPT, normofractionated proton therapy; MHPT, moderately hypofractionated proton therapy.

Changes in bowel domain scores from baseline are shown in Figure 3. In the MHPT group, no significant score deterioration from baseline was observed throughout the study. In contrast, in the NFPT group, it was observed in bowel summary, bother, and function at 12 and 36 months, although the MCID threshold was not reached. Significant differences in mean score changes between the two groups were detected for bowel summary, bother, and function at 12 months (NFPT vs. MHPT: −3.6 vs. −0.3; *p* < 0.001, −3.6 vs. −0.9; *p* = 0.003 and −3.6 vs. 0.3; *p* < 0.001, respectively) and 36 months (−2.0 vs. 0.1; *p* = 0.008, −2.2 vs. −0.2; *p* = 0.042, and −2.0 vs. 0.5; *p* = 0.003, respectively) (asterisks in Figure 2). The proportion of patients who experienced MCID in the NFPT vs. MHPT group was 37% (107/286) vs. 19% (60/314), *p* < 0.001 for bowel summary; 31% (88/285) vs. 17% (52/313), *p* < 0.001 for bother; and 37% (106/287) vs. 22% (70/316), *p* < 0.001 for function at 12 months. At 36 months, it was 29% (74/257) vs. 18% (49/270), *p* = 0.004 for bowel summary; 23% (58/256) vs. 14% (39/269), *p* = 0.018 for bother; and 32% (84/259) vs. 20% (55/271), *p* = 0.002 for function (Table 3).

Moderate to large problems in overall bowel habits were reported in 2.7% and 2.0% at baseline in the NFPT and MHPT groups, respectively. The proportion ranged from 2.1 to 3.1% until 36 months after PT, except at 12 months in the NFPT group (5.3%) (Figure 2). More patients in the MHPT group had moderate–large problems with abdominal/pelvic/rectal pain at 1 month (0% vs. 2.1%; *p* = 0.016), whereas more patients in the NFPT group had moderate–large problems with bloody stools at 36 months (3.2% vs. 0.4%; *p* = 0.018) (Table S2).

### 3.4. Sexual Domain

**Figure 4:**
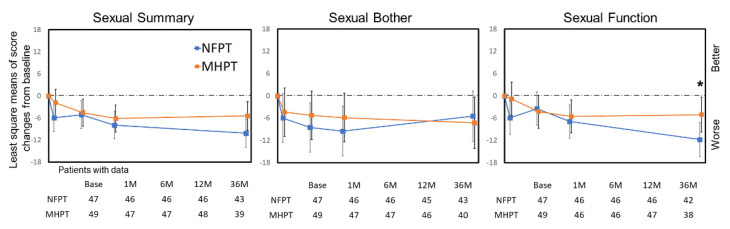
Score changes in EPIC sexual domains for patients without ADT. Least square means of score changes from baseline are plotted. Time-points represent baseline, 1, 6, 12, and 36 months, from the left. Error bars represent 95% confidence intervals. * indicate significant differences (*p* < 0.05) between NFPT and MHPT. Abbreviations: NFPT, normofractionated proton therapy; MHPT, moderately hypofractionated proton therapy.

As the sexual QOL is strongly affected by ADT usage and duration, the sexual domain of EPIC scores was analyzed only in the no ADT group (Figure 4). There was a difference in mean score changes for sexual function at 36 months between the two groups (NFPT vs. MHPT: −12.6 vs. −6.0; *p* = 0.045); mean score changes in the NFPT group for sexual bother (−9.1 points) at 6 months, sexual summary (−8.0) and bother (−9.7) at 12 months, and sexual summary (−10.5) and function (−12.6) at 36 months reached the MCID threshold (Table 3).

## 4. Discussion

This study analyzed patient-reported QOL data after MHPT for PCa in comparison with NFPT. To our knowledge, this is the largest study to present long-term QOL profiles after MHPT with relatively high questionnaire compliance rates (approximately 80% even at the 36-month follow-up). We observed small mean score changes from baseline and MCID proportions in EPIC urinary summary (−0.3 points and 21%) and bowel summary (+0.1 points and 18%) at 36 months after MHPT. Overall, this study did not reveal clinically meaningful deterioration at almost all assessment points in the urinary and bowel QOL in both NFPT and MHPT groups, and showed that MHPT had similar impacts on the urinary, bowel, and sexual QOL to NFPT over three years, which was mostly consistent with the results of large-scale randomized trials using XRT [13,14,15].

Of note, the urinary QOL temporarily decreased with clinically meaningful changes at 1 month in both groups; however, it recovered by 6 months and remained at around baseline levels until 36 months. The trend towards decline in the urinary incontinence score over time may be related to the effect of radiation and aging, since the score similarly decreased over time in patients with active surveillance [3]. The pattern of decline at 1 month and subsequent recovery is consistent with that in previous XRT or PT reports [14,22]. The urinary bother at 1 month was mainly composed of irritation and obstruction rather than incontinence (Figure 1 and Table S2). The score deterioration and MCID proportion of urinary bother were larger in the MHPT group than in the NFPT group at 1 month (−7.3 vs. −5.0 points; *p =* 0.029, and 52% vs. 42%, *p =* 0.015). This difference was in part related to the timing of data collection. Our MH and NF schedules are considered equivalent if the α/β ratio is 2.2 Gy. The α/β ratio for early mucosal reaction is usually higher than 3 Gy [28]; thus, the urinary bother scores should be higher in the MH group than in the NF group at 1 month. However, the use of a higher fractional dose or shorter overall treatment time may cause earlier acute urinary adverse events. The CHHiP trial [14] reported no difference in acute urinary toxicities between the MH and NF arms, while the peak appeared sooner in the MH arm (at 4–5 weeks after XRT start) than in the NF arm (at 7–8 weeks), and the MH arm tended to have worse urinary symptoms than the NF arm at 1 month after completion of RT. We collected the QOL data at 1 month after PT completion; therefore, urinary QOL at 1 month after MHPT might seem worse than after NFPT. Provided the responses of urothelial cells underlie acute urinary complications, the α/β ratio for urothelial cells should be further evaluated. In contrast, overall urinary QOL after MHPT was slightly better than that after NFPT at later periods; in particular, QOL of urinary summary, bother, and incontinence at 36 months after MHPT was significantly better than after NFPT. Since existing literature showed an α/β ratio for late-responding normal tissues was higher than 2.2 Gy [29], our findings observed at 36 months seemed reasonable.

A few differences between the NFPT and MHPT groups were also observed in bowel and sexual QOL. First, the bowel QOL after NFPT was similar to that after MHPT by 6 months, but was significantly poorer at 12 and 36 months, and larger proportions in the NFPT group developed moderate–large problems in bloody stools at 36 months (3.2% (8/254) vs. 0.4% (1/269); *p =* 0.018), although there was no significant difference in other specific symptoms between the groups at 12 and 36 months (Table S2). This observation could also be explained by the above-mentioned idea, because the α/β ratio for late rectal response is reported to be about 5.4 Gy [30]. However, to our knowledge, the majority of randomized trials comparing NF versus MH reported similar late gastrointestinal (GI) side effects in the two schedules or slightly worse side effects in MH [7,10,11,12], except for the PROFIT study, which reported that late grade 2+ GI toxicity increased in the NF arm [31]. Therefore, our results may be associated with an institutional learning curve effect. As we started the NFPT study first and the MHPT study later, there may have been some improvement over time in the treatment planning and daily image guidance. Indeed, we noted the incidence of rectal bleeding was higher in the NFPT group (Figure S1 and Table S3), especially among patients treated in the early period. Second, in the sexual QOL among the no ADT population, clinically meaningful deterioration was observed only in the NFPT group at several points from 6 to 36 months, although there was no significant difference in the MCID proportion between the groups. We considered it difficult to interpret the difference in potential impact on sexual QOL between NFPT and MHPT only from our data because the number of patients was small (47 patients for NFPT and 49 patients for MHPT) and there was a large variation. In general, sexual QOL is a sensitive endpoint; therefore, to investigate this issue, a better-designed examination is ideal.

The difference in QOL between post-PT and post-XRT is another concern. PT theoretically reduces low-to-moderate doses to the pelvic normal tissues compared with XRT, although high doses to neighboring organs at risk, such as the anterior rectal wall immediately posterior to the prostate, which remains unavoidable [32]. Whether this dosimetric advantage improves patient QOL remains controversial. In the CHHip trial [14], which used similar schedules to ours (NF of 74 Gy in 37 fractions and MH of 60 Gy in 20 fractions or 57 Gy in 19 fractions), moderate–large problems for overall bowel bother were reported in 5–6% of patients in each group at 24 months. On the other hand, our results showed moderate–large problems for overall bowel bother were noted in 3% (8/258) and 3% (8/269) of patients in the NFPT and MHPT groups at 36 months, respectively. In addition, we did not observe notable bowel QOL deterioration at 1 month after PT, even though it was observed in the CHHip trial at 10 weeks after RT initiation. A non-randomized study similarly reported that bowel QOL was poorer after IMRT than after PT at 2 months after IMRT and at 3 months after PT start [33]. Thus, both late-phase (two years or more) and acute-phase (within a few months after RT) bowel QOL may be better after PT than after IMRT, although comparisons should be made cautiously and these comparisons should be conducted in prospective randomized trials. We are expectantly waiting for the results of the PARTIQoL trial (NCT01617161) to make a conclusion.

In addition to patient-reported outcomes, physician-reported adverse events are shown in Figure S1 and Table S3. In general, physician-reported scoring tends to underestimate subjective (not easily observable) symptoms but is useful to evaluate objective symptoms. Our data demonstrated consistency in the results of late bowel toxicities reported from patients and physicians. This may be because the main event reducing late bowel QOL was rectal bleeding, which is a very objective and easily detectable event. By contrast, we found more frequent grade 2 acute genitourinary toxicities in the NFPT group than the MHPT group, even though QOL for urinary bother at 1 month after NFPT was better than after MHPT. Medication to relieve urinary bother such as α-blocker is often prescribed during and immediately after RT and we usually counted the adverse event requiring these medications as a toxicity. However, relief of symptoms by medications could improve the patient-reported outcomes. Since acute urinary symptoms are typically transient, it may not be problematic; however, we need to be careful when interpreting patient-reported QOL assessed in the period with frequent treatment intervention. Comprehensive observation from multidirectional approaches is important to evaluate true treatment-related toxicities.

There were several limitations to this study. First, it was non-randomized and unavoidable selection biases may exist. Another limitation was the relatively short follow-up duration. In general, evaluation of PCa treatment requires at least five years of follow-up. However, recent studies suggested two or three years to be adequate for the assessment of initial QOL because there will be little change thereafter [2,11]. We will report longer follow-up QOL results together with other endpoints in the future. Lastly, our findings will not always be applicable to all PCa patients because QOL change is a sensitive endpoint easily affected by many factors (e.g., age, lifestyle, personality, comorbidity, and baseline symptoms); thus, more subdivided analyses according to baseline patient characteristics with a larger study population may be helpful for personalizing the information given to individual pretreatment patients.

## 5. Conclusions

Our study demonstrated that MHPT had small negative impacts on urinary, bowel, and sexual QOL over three years, and had similar impacts on the QOL to NFPT in general, although sexual QOL should be further evaluated with a larger number of patients. Hypofractionation is more convenient for patients and improves resource utilization. Our findings may be valuable for counseling in the patient decision-making process, although additional assessments with a longer follow-up are required to evaluate the overall clinical value of MHPT.

## Figures and Tables

**Table 1:** Patient, tumor, and treatment characteristics.

Characteristics	NFPT (*n* = 296)	MHPT (*n* = 343)	*p* Value
Age, median years (range)	69 (51–90)	69 (46–86)	0.16
T stage (%)			0.64
T1/T2/T3	72 (24)/178 (60)/46 (16)	73 (21)/217 (63)/53 (16)	
Gleason score (%)			0.15
6/7/8–10/Unknown	80 (27)/116 (39)/100 (34)/0 (0)	80 (23)/160 (47)/101 (29)/2 (1)	
Initial PSA, ng/mL			0.016
Median (IQR)	9.0 (6.1–14.8)	7.6 (5.6–12.3)	
Mean (SD)	14.3 (17.1)	13.6 (20.7)	
NCCN risk group (%)			0.19
Low/Intermediate/High	50 (17)/117 (40)/129 (43)	51 (15)/160 (47)/132 (38)	
Prostate volume, cc			0.23
Median (IQR)	28.6 (23.4–37.1)	26.6 (22.1–35.8)	
Mean (SD)	32.1 (12.7)	30.9 (14.1)	
CTV, cc			0.25
Median (IQR)	31.5 (25.6–40.3)	29.3 (24.7–38.6)	
Mean (SD)	34.6 (12.7)	33.4 (13.9)	
Hypertension (%)			0.18
Yes	69 (23)	97 (28)	
Diabetes (%)			0.55
Yes	34 (11)	45 (13)	
ADT (%)			0.16
None/N/N-A	47 (16)/121 (41)/128 (43)	49 (14)/166 (48)/128 (37)	
PT technique (%)			<0.001
Passive scattering/Spot scanning	264 (89)/32 (11)	342 (99.7)/1 (0.3)	

Abbreviations: NFPT, normofractionated proton therapy; MHPT, moderately hypofractionated proton therapy; PSA, prostate-specific antigen; NCCN, National Comprehensive Cancer Network; CTV; clinical target volume; ADT, androgen deprivation therapy; N, neoadjuvant; N-A, neoadjuvant-adjuvant.

**Table 2:** Baseline EPIC scores.

EPIC Domain	NFPT (*n* = 296)		MHPT (*n* = 343)		*p* Value
	Mean Score (Range)	SD	Mean Score (Range)	SD	
Urinary					
Summary	92.2 (51.4–100)	8.7	91.8 (41.0–100)	9.5	0.51
Bother	89.4 (42.9–100)	11.8	88.6 (32.1–100)	12.5	0.43
Function	96.4 (53.4–100)	7.4	96.2 (41.8–100)	8.3	0.77
Incontinence	95.2 (48.0–100)	10.2	94.9 (22.8–100)	11.6	0.63
Iritative/obstructive	92.0 (53.6–100)	9.3	91.7 (39.2–100)	9.9	0.72
Bowel					
Summary	93.5 (42.9–100)	8.4	93.7 (53.6–100)	7.2	0.69
Bother	94.9 (32.1–100)	9.3	95.5 (42.9–100)	7.8	0.35
Function	92.1 (46.4–100)	8.9	92.0 (50.0–100)	8.7	0.83
Sexual Summary					
All patients	33.7 (0–75.0)	10.8	34.1 (2.5–85.4)	10.7	0.68
ADT (-)	46.8 (15.4–75.0)	14.3	46.7 (21.2–85.4)	16.3	0.98
Sexual Bother					
All patients	90.6 (0–100)	19.8	91.2 (0–100)	18.7	0.68
ADT (-)	86.4 (25–100)	18.7	88.5 (31.3–100)	17.7	0.58
Sexual Function					
All patients	8.3 (0–62.5)	14.9	8.6 (0–78.1)	14.9	0.79
ADT (-)	28.8 (0–62.5)	18.7	28.4 (0–78.1)	22.4	0.93

Abbreviations: NFPT, normofractionated proton therapy; MHPT, moderately hypofractionated proton therapy; EPIC, Expanded Prostate Cancer Index Composite; ADT, androgen deprivation therapy.

**Table 3:** Mean score changes and proportion of minimum clinically important differences at 1, 6, 12, and 36 months.

MSC and MCID at 1–36 Months		1 Month							6 Months						
		NFPT			MHPT				NFPT			MHPT			
EPIC Domain	1/2 SD ^a^	*n*	MSC ^b^	MCID (%)	*n*	MSC ^b^	MCID (%)	*p* Value ^c^	*n*	MSC ^b^	MCID (%)	*n*	MSC ^b^	MCID (%)	*p* Value ^c^
Urinary															
Summary	4.6	290	−5.0	122 (42)	327	−6.2	161 (49)	0.076	289	−1.3	74 (26)	313	0	65 (21)	0.18
Bother	6.1	290	−5.0	121 (42)	327	−7.3	169 (52)	0.015	289	−1.0	80 (28)	313	0	79 (25)	0.52
Function	4.0	290	−4.8	105 (36)	329	−4.9	140 (43)	0.12	290	−1.9	69 (24)	314	−0.1	60 (19)	0.17
Incontinence	5.5	286	−3.6	71 (25)	327	−3.9	93 (28)	0.32	288	−0.7	51 (18)	311	0.7	39 (13)	0.086
Irritative/obstructive	4.8	290	−5.3	119 (41)	327	−6.8	150 (46)	0.26	289	−1.5	68 (24)	313	−0.4	69 (22)	0.70
Bowel															
Summary	3.9	292	−0.2	71 (24)	330	−0.3	65 (20)	0.17	293	−0.3	70 (24)	315	0	60 (19)	0.17
Bother	4.3	292	−0.1	58 (20)	330	−0.6	50 (15)	0.14	293	−0.4	50 (17)	315	−0.8	60 (19)	0.60
Function	4.4	293	−0.3	69 (24)	331	−0.1	73 (22)	0.70	293	−0.3	66 (23)	315	0.7	62 (20)	0.43
Sexual (no ADT)															
Summary	7.6	46	−5.8	12 (26)	47	−2.1	14 (30)	0.82	46	−5.6	17 (37)	47	−4.5	17 (36)	1
Bother	9.0	46	−6.5	13 (28)	47	−4.7	12 (26)	0.82	46	−9.1	17 (37)	47	−5.5	14 (30)	0.51
Function	10.2	46	−5.4	13 (28)	46	−1.0	13 (28)	1	46	−3.9	14 (30)	46	−4.1	14 (30)	1
**MSC and MCID** **at 1–36 Months**		**12 Months**							**36 Months**						
		**NFPT**			**MHPT**				**NFPT**			**MHPT**			
**EPIC Domain**	**1/2 SD ^a^**	***n***	**MSC ^b^**	**MCID (%)**	***n***	**MSC ^b^**	**MCID (%)**	***p*** **Value ^c^**	***n***	**MSC ^b^**	**MCID (%)**	***n***	**MSC ^b^**	**MCID (%)**	***p*** **Value ^c^**
Urinary															
Summary	4.6	284	−1.9	74 (26)	314	−1.1	78 (25)	0.78	259	−1.6	62 (24)	267	0.0	56 (21)	0.47
Bother	6.1	284	−1.4	83 (29)	315	−1.1	83 (26)	0.47	259	−0.7	73 (28)	266	1.1	49 (18)	0.010
Function	4.0	286	−2.7	76 (27)	316	−1.3	70 (22)	0.22	260	−2.6	67 (26)	269	−1.3	56 (21)	0.18
Incontinence	5.5	284	−2.2	61 (21)	315	−0.6	57 (18)	0.31	256	−3.6	65 (25)	260	−1.5	49 (19)	0.089
Irritative/obstructive	4.8	284	−1.7	71 (25)	314	−1.4	71 (23)	0.50	259	−0.4	56 (22)	266	0.7	47 (18)	0.27
Bowel															
Summary	3.9	286	−3.6	107 (37)	314	−0.3	60 (19)	<0.001	257	−2.0	74 (29)	270	0.1	49 (18)	0.004
Bother	4.3	285	−3.6	88 (31)	313	−0.9	52 (17)	<0.001	256	−2.2	58 (23)	269	−0.2	39 (14)	0.018
Function	4.4	287	−3.6	106 (37)	316	0.3	70 (22)	<0.001	258	−1.9	82 (32)	272	0.5	56 (21)	0.002
Sexual (no ADT)															
Summary	7.6	46	−8.0	23 (50)	48	−6.1	19 (40)	0.41	43	−10.5	26 (60)	39	−5.9	19 (49)	0.38
Bother	9.0	45	−9.7	16 (36)	46	−6.0	18 (39)	0.83	43	−5.5	14 (33)	40	−7.3	17 (43)	0.37
Function	10.2	46	−6.9	18 (39)	47	−5.5	11 (23)	0.12	42	−12.6	24 (57)	38	−6.0	15 (39)	0.13

Abbreviations: NFPT, normofractionated proton therapy; MHPT, moderately hypofractionated proton therapy; EPIC, Expanded Prostate Cancer Index Composite; SD, standard deviation; MSC, mean score change; MCID, minimum clinically important difference; ADT, androgen deprivation therapy. ^a^ = 1/2 SD for each domain summary or subscale baseline score in all patients, and in patients without ADT (for sexual domain only). ^b^ = Mean score changes from baseline to each time point. ^c^ = Comparison of proportion of MCID between NFPT and MHPT. These *p* values were calculated by the Fisher’s exact test.

## Data Availability

The data presented in this study are available on request from the corresponding author. The data are not publicly available due to institutional guidelines.

## Supplementary Materials

The following are available online at https://www.mdpi.com/article/10.3390/cancers14030517/s1, Figure S1: Cumulative incidence of toxicity; Table S1: Dose constraints for NFPT and MHPT, Table S2: Response to specific questions in EPIC, Table S3: Acute and late toxicity.
