# Supplementary material for: Patient-Reported Quality of Life Outcomes after Moderately Hypofractionated and Normofractionated Proton Therapy for Localized Prostate Cancer
Source: Cancers (Basel). 2022 Jan 20;14(3):517. doi: 10.3390/cancers14030517 (PMC8833499; doi:10.3390/cancers14030517)
Supplement: Supplementary file 1 [file cancers-14-00517-s001.zip › cancers-1532821-supplementary.pdf]

cancers

Supplementary Materials

Patient-Reported Quality of Life Outcomes after Moderately Hypofractionated and Normofractionated Proton Therapy for Localized Prostate Cancer

Koichiro Nakajima [1,*], Hiromitsu Iwata [1], Yukiko Hattori [1], Kento Nomura [1], Kensuke Hayashi [2], Toshiyuki Toshito [3], Yukihiro Umemoto [4], Shingo Hashimoto [5], Hiroyuki Ogino [1] and Yuta Shibamoto [5,6]

1 Department of Radiation Oncology, Nagoya Proton Therapy Center, Nagoya City University West Medical Center, Nagoya 462-8508, Japan; h-iwa-ncu@nifty.com (H.I.); hattoriy@med.nagoya-cu.ac.jp (Y.H.); knomura01i04m08t@gmail.com (K.N.); hogino2@med.nagoya-cu.ac.jp (H.O.)
2 Department of Proton Therapy Technology, Nagoya Proton Therapy Center, Nagoya 462-8508, Japan; k.hayashi.12@west-med.jp
3 Department of Proton Therapy Physics, Nagoya Proton Therapy Center, Nagoya 462-8508, Japan; t.toshitou.20@west-med.jp
4 Department of Urology, Nagoya City University West Medical Center, Nagoya 462-8508, Japan; uro-ume@med.nagoya-cu.ac.jp
5 Department of Radiology, Nagoya City University Graduate School of Medical Sciences, Nagoya 467-8601, Japan; hashimoto.ncu@gmail.com (S.H.); yshiba@med.nagoya-cu.ac.jp (Y.S.)
6 Department of Radiation Oncology, Narita Memorial Proton Center, Toyohashi 441-8021, Japan
* Correspondence: km_rad@med.nagoya-cu.ac.jp; Tel.: +81-52-991-8577

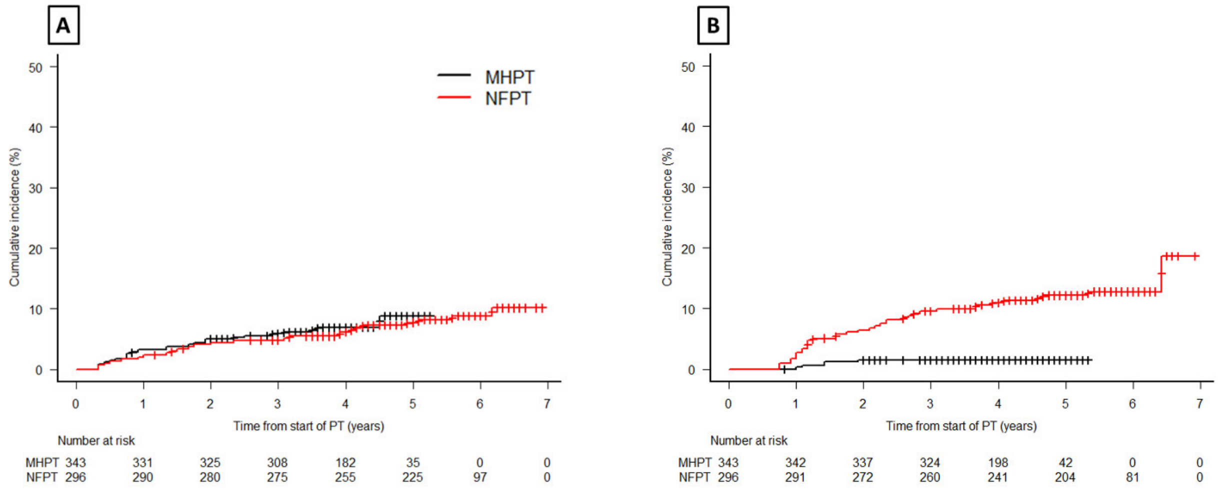

Figure S1. Cumulative incidence of (**A**) grade 2 or higher late genitourinary toxicity and; (**B**) grade 2 or higher late gastrointestinal toxicity. The estimated cumulative 3-year incidence of late grade 2 or higher genitourinary and gastrointestinal toxicity in the NFPT vs MHPT group was 4.8% (95% CI, 2.8% to 7.9%) vs. 5.9% (3.8% to 8.9%), $p = 0.66$, and 9.5% (6.7% to 14%) vs. 1.5% (0.6% to 3.5%), $p < 0.001$.

Table S1. Dose constraints for organs at risk.

	NFPT		MHPT
**Total Dose (Gy (RBE))**	**74 78**		**60 63**
Rectum Vx		Rectum Vx	
65	15%	53.5	15%
60	18%	50	18%
40	30%	34.5	30%
Bladder Vx		Bladder Vx	
65	25%	54.5	25%
40	50%	34.5	50%
Small bowel Dmax	55 Gy (RBE)		46 Gy (RBE)
Large bowel Dmax	61 Gy (RBE)		51 Gy (RBE)
Hip joint Dmax	50 Gy (RBE)		42.5 Gy (RBE)

Abbreviations: NFPT, normofractionated proton therapy; MHPT, moderately hypofractionated proton therapy; Gy (RBE), gray relative biological effectiveness equivalents. Vx = percentage of the organ receiving at least X Gy (RBE).

Table S2. Response to specific questions in EPIC.

**Proportion of Patients Who Complained of a "Moderate" to "Big" Problem at Each Follow-up Point, among "No" to "Small" Problem at Baseline.**												
	**1 Month**			**6 Months**			**12 Months**			**36 Months**		
	**NFPT (%)**	**MHPT (%)**	***p* value**	**NFPT (%)**	**MHPT (%)**	***p* value**	**NFPT (%)**	**MHPT (%)**	***p* value**	**NFPT (%)**	**MHPT (%)**	***p* value**
Urinary: How big a problem, if any, has each of the following been for you during the last 4 weeks?												
Dripping or leaking urine	6 (2.1)	4 (1.2)	0.53	2 (0.7)	2 (0.7)	1	7 (2.5)	1 (0.3)	0.031	6 (2.3)	0 (0)	0.014
Pain or burning on urination	10 (3.4)	9 (2.8)	0.65	6 (2.1)	3 (1.0)	0.33	6 (2.1)	6 (1.9)	1	3 (1.2)	3 (1.1)	1
Bleeding with urination	1 (0.4)	1 (0.3)	1	0 (0)	1 (0.3)	1	3 (1.1)	2 (0.6)	0.67	1 (0.4)	1 (0.4)	1
Weak urine stream or incomplete emptying	14 (5.0)	24 (7.4)	0.24	8 (2.9)	7 (2.3)	0.80	10 (3.6)	12 (3.9)	1	8 (3.2)	6 (2.3)	0.60
Waking up to urinate	27 (10)	30 (10)	1	15 (5.7)	9 (3.2)	0.21	16 (6.2)	12 (4.3)	0.34	19 (8.2)	9 (3.8)	0.051
Need to urinate frequently during the day	21 (7.6)	40 (13)	0.04	13 (4.7)	15 (5.2)	0.85	12 (4.4)	16 (5.5)	0.70	10 (4.1)	5 (2.0)	0.2
Bowel: How big a problem, if any, has each of the following been for you?												
Urgency to have a bowel movement	3 (1.1)	4 (1.2)	1	4 (1.4)	4 (1.3)	1	4 (1.4)	4 (1.3)	1	6 (2.3)	3 (1.1)	0.33
Increased frequency of bowel movements	4 (1.4)	5 (1.5)	1	7 (2.4)	4 (1.3)	0.37	6 (2.1)	3 (1.0)	0.32	3 (1.2)	2 (0.8)	0.68
Watery bowel movements	3 (1.1)	1 (0.3)	0.35	2 (0.7)	3 (1.0)	1	0 (0)	5 (1.6)	0.063	2 (0.8)	4 (1.5)	0.69
Losing control of your stools	1 (0.4)	0 (0)	0.47	0 (0)	5 (1.6)	0.063	2 (0.7)	4 (1.3)	0.69	2 (0.8)	3 (1.1)	1
Bloody stools	0 (0)	1 (0.3)	1	4 (1.4)	6 (1.9)	0.75	15 (5.3)	10 (3.2)	0.23	8 (3.2)	1 (0.4)	0.018
Abdominal/Pelvic/Rectal pain	0 (0)	7 (2.1)	0.016	2 (0.7)	4 (1.3)	0.69	3 (1.1)	2 (0.7)	0.67	2 (0.8)	4 (1.5)	0.69

Abbreviations: EPIC, Expanded Prostate Cancer Index Composite; NFPT, normofractionated proton therapy; MHPT, moderately hypofractionated proton therapy.

Table S3. Acute and late toxicity.

	**Genitourinary**			**Gastrointestinal** [a]		
	**NFPT (*n* = 296)**	**MHPT (*n* = 343)**	***p*** [b]	**NFPT (*n* = 296)**	**MHPT (*n* = 343)**	***p*** [b]
Acute Grade 2	47 (16%)	26 (8%)	0.001	0	1 (0.3%)	1
Acute Grade 3	0	0		0	0	
Late Grade 2	21 (7%)	24 (7%)	0.66	33 (11%)	4 (1%)	<0.001
Late Grade 3	4 (1%)	1 (0.3%)		4 (1%)	1 (0.3%)	

Scored with the modified Common Terminology Criteria for Adverse Events, version 4.0, in which prescribing two and more types of drugs for dysuria was counted as a grade 2 genitourinary toxicity. Acute toxicities were defined as those occurring within 3 months of the start of proton therapy. [a] = All gastrointestinal toxicities were proctitis or rectal bleeding. [b] = Comparison of proportion of grade 2 or higher toxicities between NFPT and MHPT. These *p* values were calculated by the Fisher's exact test.
